# Case management for the elderly with complex needs: cross-linking the views of their role held by elderly people, their informal caregivers and the case managers

**DOI:** 10.1186/s12913-016-1892-6

**Published:** 2016-11-08

**Authors:** Frédéric Balard, Marie-Christine Gely-Nargeot, Aline Corvol, Olivier Saint-Jean, Dominique Somme

**Affiliations:** 1 Laboratoire Lorrain de sciences sociales, Université de Lorraine, Nancy, France; 2 Epsylon (EA 4556), Université de Montpellier, Montpellier, France; 3 Centre Hospitalier de Rennes, Service de Gériatrie, Rennes, France; 4 Hôpital Européen Georges-Pompidou, Service de Gériatrie, Paris, France; 5 Université de Rennes 1, Centre Hospitalier de Rennes, Service de Gériatrie, Laboratoire CRAPE Centre de Recherches sur l’Action Politique en Europe, UMR 6051 Rennes, France

**Keywords:** Alzheimer’s disease, Case management, Frail older people, Old people with complex needs, Patient’s experience, Qualitative method

## Abstract

**Background:**

In 2009, case management interventions were a new social service in France implemented within the framework of the PRISMA-France program (2006–2010). People who had benefitted from case management intervention were individuals, over 60 years old living at home in situations deemed complex by professionals. Their informal caregivers were also considered as users of the service. This research accompanied the interventions during the implementation of PRISMA-France attempting to explore the users’ (old people and their informal caregivers) and case managers’ first experiences of case management. Its aim is to provide a thorough knowledge of the dispositive in order to reveal any initial failings and to ensure optimum conditions for the onset of full implementation.

**Methods:**

The study had a qualitative explorative design. Cross-linked representations of case-management were obtained through opened-ended and guided interviews with three types of informants: old people (19), their informal caregivers (11) and the case managers (5) who participated in the program during the first 6 months. Thematic analysis of the data was carried out.

**Results:**

The analysis revealed that each group of people generated its own representations of the case manager’s role, even though the three groups of informants shared the idea that the case manager is first and foremost a helper. The case managers insisted on their proximity to the old people and their role as coordinators. The informal caregivers saw the professional as a partner and potential provider of assistance in accompanying old people. The old people focused on the personal connections established with the case manager.

**Conclusion:**

The innovative and experimental dimension of case management in France and the flexible nature of the role generated a broad spectrum of representations by those involved. These different representations are, in part, the fruit of each individual’s projected expectations of this social service.

Analyzing the first representations of the case manager’s role during the implementation phase of the CM service appears as a necessary step before considering the effects of the services. The implementation and the success of a case management model have to be evaluated regarding the previous healthcare context and the expectations of the people involved.

## Background

Between 2006 and 2010, the PRISMA-France project studied the possibility of implementing in France a model of integrated social services [1] previously tested successfully in Québec [2].

The case-management model (CM) used can be defined as a “targeted, community-based and proactive approach to care that involves case-finding, assessment, care planning, and care co-ordination” [3]. Considering the heterogeneity inherent in case management, the model is classically identified as corresponding to “clinical case management” [4], in which “a designated care manager combines planning and coordination with a therapeutic, supportive role” [5]. Case management as introduced into France can thus be qualified as clinical and intensive, according to Challis’s criteria [6].

The aims of the whole PRISMA research protocol were (1) to measure the implementation of the model, (2) to observe the modifications of professional practice and (3) to explore the users’ (old people and their informal caregivers) and case managers’ experiences of case management. This paper is focused on the former’s representations; it means those which were collected during the first months of the project, we called the implementation phase.

As is showed by the literature, there is a need for in-depth investigations to gain deeper understanding of the interventions. With reference to Sandberg et al. [7] we consider that “Qualitative studies are important to identify different barriers and facilitators that could be underlying reasons for an intervention being successful or not and are necessary for implementation”. At the beginning of a CM program, pre-conceptions and clichés could be barriers for implementation that is why, before regarding the outcomes of the CM service on users’ (old people and their informal caregivers) everyday life, the first step was to analyze their representations of the service. The multiple “roles” given to the case manager by the users such as “coaching guard”, “helping hand”, “coach” [8], “navigator” [9, 10], “friend” [11], have already been discussed in the literature. These words reveal the aspect of the work of the case manager on which the interviewee is focused. Some research has demonstrated that the role of the case manager is not immediately understood by people Brubaken et al. [12] and is sometimes perceived as useless. The representation and conception of the case manager’s role is a key point of the intervention as it has an influence in several areas: the type of interaction between the case manager and the users, the expectations of the users with regard to the work of the case manager, the subjective evaluation of the success of the intervention and the feeling that the job has been correctly be done.

We will now describe and analyze the representations of the CM given by three kinds of informants: old people, their informal caregivers and the case managers themselves. The aim is to better understand the significations of the labels used by each type of informant in the French context and to trace the features (shared or not) of their representation of the case manager and her/his role.

## Methods

The study had an inductive qualitative design and can be characterized as grounded theory [13], following Kaufmann’s protocol [14].

### Participants

We considered three types of “participants”: old people, their informal caregivers and case managers. The old people and their informal caregivers, called users, are the main receivers of the potential benefits of the CM service. The case managers are in charge of the assessment of old people’s needs and the coordination of the care plan.

All the old people and their informal caregivers included in CM service of PRISMA-France project were potentially included in the research protocol. After describing the inclusion criteria of the CM service we will go on to explain why some were not included in the research.

The old people benefiting from the CM service were people over 60 years old selected because of the complexity of maintaining their autonomy at home. The evaluation of the level of complexity was not induced by researchers but estimated by healthcare professionals who were essentially nurses, GP and social workers. We argue that complexity is a fluctuating concept that could change with regard to the local context. That is why we affirm that professionals were in the best position to consider whether a situation was complex or not.

For them, complexity originated from the accumulation of several problems: cognitive impairment, regardless of its cause, a serious (chronic, sudden or chronic) health problems resulting in loss of autonomy, but also diverse psychiatric disorders, social problems (e.g. debt, risk of eviction from their home, conflict with neighbors, social isolation) and the number of old people refusing care [15]. Among the 40 people included during the implementation phase, 24 were considered as refusing care by professionals

We explained in a previous paper how the fieldwork was done [16]. All of the old people who benefit from case management were not included in the research. Ten had begun using the case management services too recently, five were temporarily or permanently not living in their homes (because of hospitalization), three had refused (at that time) a case manager’s assistance and one each was completely deaf, did not speak French, was either unable to sign the written consent form or refused to do so.

As for most of the sample, cognitive impairments or dementia were only assumed and the diagnosis of Alzheimer disease had not been established but we only include the old people who were able to discuss and give their consent to participate to the study. In agreement with Cotrell [17], Cowdell [18], Hellström et al. [19], we do not consider that cognitive impairments are a sufficient for exclusion What is more, the literature proves that people with cognitive impairments or dementia are able to give their feelings and representations about the care they receive [20]. Failure to take their opinions into consideration leads to social stigma and would mean that the point of view of the most representative profile of case management (frail old people) would have been ignored. The interviewer/researcher had respected the will of old people to participate or not to the study. The old people assumed and diagnosed “with Alzheimer disease” have signed the informed consent form, sometimes after questioning the interviewer/researcher and/or the informal caregiver when he/she was present. The interviewer/researcher was careful that he or/and the informal caregivers did not influence the old people and considered that if the participant wanted to participate and said that he/she had understood the protocol, it would not be justifiable to exclude them. To respect the value of old people’s consent, no additional consent form was asked to the informal caregiver or kin even if they gave verbally their consent. Of course, the endorsement of the informed consent was not considered by the interviewer to constitute definitive approval until the end of the fieldwork. The researcher kept in mind that the participant could “exercise” their autonomy [21] and express their refusal to participate at any time during the interview. One person refused to sign even though she accepted the interview and the tape recording, was not included in the research.

The informal caregivers involved in the research were people recognized as close helper by the old people. Their identity does not necessarily correspond to the name of the official caregivers given in administrative forms. Sometimes old people gave the name of their son in official forms but explained to the social worker that they did not see him more than once a year and thus considered that the person to contact initially was the neighbor or their guardian.

Considering their supportive role and the difficulties and suffering these informal caregivers mentioned, they were also the target of the CM program that is why their representations needed to be considered. The 11 informal caregivers have different profiles: a mother and her daughter living in the same home as the case-management user; two were husbands caring for their spouses; two were guardians of the buildings in which the old people lived; two were neighbors caring for the same user; three were daughters of old people who had very different profiles: one who took great care of her parents but did not live with them, another lived more than 200 km from her mother but visited twice a month; and the last lived in the same town as her mother with whom she was in conflict.

Five women, all of whom were included in the study, were employed as case managers at the time of the inquiry. Their initial training varied: two were social workers, two nurses and one a psychologist (cf. Table 1). All had followed the same academic training originally provided on an experimental basis which has since become the university diploma required to become a case manager in France. These professionals work closely with the user’s doctor, even though (or even when) the latter did not prescribe the onset of case management. They were included in the study because it appeared necessary to see how they feel about their new profession as this could influence their practice.

**Table 1:** Type of informants and the number of interviews conducted

Type of informant	Number of interviews			
	1	2	3	Total
Old people	17	1	1	19
Informal caregiver	6	4	1	11
Case manager	5	0	0	5
Total	28	5	2	35

This methodology has an emic posture approach that aims to take into account the participants’ subjectivity as a real source of data. Accordingly, case managers, old people and their informal caregivers, were considered as “meaning makers” [22] of the case management program.

### Ethical considerations

The French Committee for the Protection of Human Research Subjects for Île-de-France has given its approval for this research classified as non- interventional (“*recherche non interventionelle*”) and non-biomedical research. All the participants involved in this research read the information letter and signed the informed consent form.

### Data collection

The interviews used in this paper were conducted during the first 6 months of the case management program, i.e. the implementation phase.

The inquiry consisted of two phases: the first, called *comprehensive* [14], took the form of an open-ended interview; and the second, a guided interview. With the case managers and some of the other informants these two phases were completed during the same interview. For several others (see Table 1), the old people and their kin and neighbors were seen two or three times to give more substance to the inquiry. To enhance quality in data collection and avoid disparities, all the interviews were conducted face-to-face, recorded and transcribed by the first author who is an anthropologist experienced in conducting interviews with fragile, elderly subjects [23]. We agree with Cowdell [18], that the researcher is the major instrument of data collection and quality, in particular with old people with dementia.

The comprehensive part of the interview sought to obtain the representations and life experiences of the informants without influencing their responses. A general open question was asked of the old people: “Since (number of week since the beginning of CM service) weeks, you have been receiving the visit of (family name or surname of the case manager), could you tell me about that?

Then, the researcher/interviewer only used short words/questions to keep the interview going, mostly by rephrasing the words of the informants, like “so you said that you appreciate her visits, could you tell me why?”; “you said that she is there to help you, what do you understand about her role?”; “When you said: ‘she is efficient’, want do you mean, could you give me examples?”.

The opened-ended interview used for the informal caregivers began with a first general question: “Since (number of week since the beginning of CM service) weeks, (name or relationship with the proxy) receive the visit of (family name or surname of the case manager), could you tell me about that?

Then the informal caregiver was asked to develop on what she/he noted and understood about the role of the case manager. For the case manager, the unstructured part of the interview began with the question: “You have been recruited as case manager since (number of months) months, what could you tell me about your job? Then, the case managers were asked to describe and explain what they consider as part of their role.

The guided interviews were semi-structured and were oriented in two directions: (1) the development of what the informants said during the unstructured part; (2) the opening up of themes that are considered as the main hypothesis as generated by the literature review or the first interviews collected. The order and formulation of the questions were not fixed as the order depended of the flow of the interview.

In this part of the study, the old people were asked to describe a typical day in order to gain an idea of what they saw as their needs and difficulties. They were then asked to describe as precisely as possible what the case manager had done with and for them. Another theme concerned the type of relationship built with the case manager. Finally, the interviewer/researcher asked what they considered as positive and negative in the intervention and role of the case manager.

The main themes investigated with the informal caregivers were their representations of the situation and their role in it. They were asked to give their opinion on the role of the case manager and what they thought about her interventions.

The case managers were asked to explain their conceptions of their role and work the nature of their interventions and their relationships with the users and especially with old people were considered as particularly important. They were also asked what had motivated them to become a case manager and to go into detail about their expectations. Finally, the interviewer/researcher asked them to say what they thought were the successes and limits of their interventions.

### Analysis

The comprehensive interviews were analyzed as explained above using Nvivo software. The framework for the analysis was built using the informants own words in an open-coding model. As the analysis was conducted in parallel with to the fieldwork it could be used as feedback for the semi-directive interviews. Analysis of the *verbatim* texts was discussed among the multidisciplinary team members including the interviewer/researcher, a sociologist and a geriatrician. The analysis was carried out in two stages. Firstly, each verbatim was analyzed in its own right. The aim of this first analysis was to expose the main dimension of the experience of the case management for the informants. This also permitted to review the guided interview and to generate new hypotheses.

A second type of analysis was done by comparing the *verbatim* via a thematic analysis. This permits a deep study the point of view of the informants on the main themes generated by the fieldwork.

The *verbatim* from interviews with old people diagnosed or potentially suffering from Alzheimer’s disease were not analyzed differently from the others. Discourse was interpreted in context, taking into account the interaction with the researcher, the life story elements known and also the data coming from the interviews made with case managers and informal caregivers. The main aim was not to check the validity of what they said even if we always crossed the data between informants, but to understand how they live what they feel about it, and what it could tell us about the implementation phase. By focusing on representations as sufficient phenomena to explore the strengths and weaknesses of the implementation phase, we avoided the question of recall bias or memory loss.

## Results

**Fig. 1:**
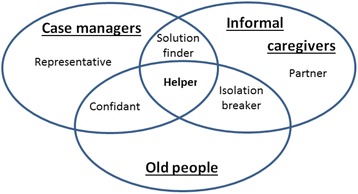
Diagram showing case-manager representations according to the informant: case manager, user or informal caregivers

Figure 1 gives an illustration of the crossing of viewpoints of the CM by our informants.

### What case managers say about their role

The analysis of interviews enabled us to organize the case mangers’ thoughts across three major dimensions: interaction between the case manager and the old people, their vision of the case manager’s role in the follow-up of the situation, and the interactions with other professionals and the informal caregivers.

#### The case manager as representing the old people

All the case managers first mentioned the interaction with the elderly user as the essential component of their work. Two of them used the term “*référent*”, in a way comparable to “representative”: “I would define it as the role of *référent*, representing the person (…). That means that a certain proximity to the person is needed…”

The case managers agreed with the idea that their role was to be at the fragile elderly person’s side: “My role is to be a facilitator of the administrative processes and a kind of companion and helper, a type of coach or guide for the different persons.” Two of them used the term “companion”. This idea contained several nuances, ranging from the simple presence to supervision or orientation. The case manager who used the term guide and coach said that she has to be a “facilitator”. The word “help” was also used, as was “support”.

We also encountered the idea that this job implies being the spokesperson for the elderly client, with one of the case managers even using the term “defender”. The role is to overcome incapacities or impairments which may hamper the expression or autonomy of the elderly persons by becoming a medium via whom the user expresses him/herself and his/her choices. In this framework, case managers frequently used the terms of “listen”, “confidence” and “proximity”.

#### A confidant

During the interviews, the case managers insisted on their role of confidant (“*confident*”) They described this role with reference to the concept of “needs, considering that "answering the need" is the main role of the case manager. This aspect took priority over the CM's interventional role (evaluating, planning and coordinating). At this stage, the concrete demonstration of their actions was still unclear. “Enabling the aged individual to remain at home under the best possible conditions, therefore being able to recognize his/her needs…” They insisted on the means available to them by highlighting the ideas of “availability” and “intensity”. “Having the time” and, thus, being available was presented by the case managers as a fundamental aspect of their role. The multiplicity of visits and the time spent with the user, and being attentive to each one’s needs created a new form of “intimacy”.

#### A solution finder

The third dimension corresponds to the interactions between the informal caregivers and the professionals. One case manager defined herself as a “centralizer and distributor” of information and another explained that part of her work consisted of “reformulating” the user’s needs to participating professionals. These comments highlight the dual interface activity that the case managers recognized as their role: from user to system and vice versa. The case managers again positioned themselves as facilitators to the extent that they collected a variety of information concerning the user’s situation and transmitted it to the appropriate person(s). This reflected the “coordinating function” that they all claimed as theirs, saying they had to “sort the information” they collected, but also to translate it into an “intelligible format” for each of the people involved in the situation. The case manager sometimes played the role of “conciliator”, and became a “buffer” between users and professionals, and even between the old people and their informal caregiver. These last two terms revealed their rapid awareness of the possible tensions between users and professionals. The high number of old people refusing assistance and care at the onset of the program undoubtedly explains, in part, this representation. That the old people’s informal caregiver was rarely mentioned could reflect the isolation of many of them at this stage of the program.

### The informal caregivers opinions of the case manager’s role

The different informal caregiver profiles explained how their representations and expectations of case management could vary widely. Nevertheless, they agreed on some major points.

#### A partner

Confronted with problems to which they could not find solutions, the informal caregivers have great difficulty resolving the situations they are experiencing. They described the case manager as the one that “handles the problems” and finds ways of dealing with difficulties and emphasized the partnership established with the case manager. One user’s daughter explained that she benefitted from a personalized relationship and regular exchanges by phone with the case manager. The case manager was kept informed of the progress of the proceedings, the successful solutions that she had found, while the daughter called when a particular problem arose. Another daughter explained that the case manager’s intervention helped her de-dramatize situations and to no longer feel isolated when faced with difficulties.

#### A solution finder

Unlike the caregiving daughters, some neighbors and, to an even greater extent, the two guardians explained they had become involved despite themselves in the caregiving relationship. For them, the case manager was the professional who would enable them to stand back and unload certain responsibilities. The daughter living 200 km away from her mother saw the case manager as the professional serving as a link between her and her mother who helped her resolve “from afar” problems which were much too complicated for her to cope with.

Many of these informal caregivers mentioned the case managers’ ability to understand the problems and to find appropriate solutions. They said that the case manager was a “decision-maker” who was responsible for the situation. Here again, the case manager was described as a “*réferent*”, to whom one refers to make the right decision. “She is my person of reference, my *«réferent*”. When things are not going well, I tell her. (…) You need someone to contact in times of need. (…) we take the advice of (case manager’s name), she is the one to say yes or no, what can or cannot be done.”

#### An isolation breaker

The informal caregivers’ representations of the case managers focused on the difficulties and problems and their resolution. They insisted on their feeling isolation and solitude. For them, the case manager is a partner and an isolation breaker. One of the neighbors did not consider that as positive at first, viewing the case manager’s intervention as lost time which would have been better devoted to more assistance and care. Worse, she considered that it complicated the accompaniment: “I said, what a waste of our time… on forms, telephone calls, while I thought that she should be out in the field.” Several meeting were necessary for the neighbor to understand that the case manager cannot come everyday to perform care and housework but rather that they were a person who could evaluate the situation and coordinate the intervention of healthcare professionals.

### Old people talk about the case manager

Old people benefitting from case management were characterized as being individuals with unmet needs, isolated and often dissatisfied with the aid proposed by professionals. The clarity lacking about the case manager’s role led these old people to place many of their expectations in her that do not fall within the usual scope of assistance and care. Thus, they sought from the case manager what they could expect from a friend: someone to keep them company, who is there to listen to them and, finally, to defend them or their opinions.

Due to the physical and mental impairments of the old people the case managers often have difficulties explaining their tasks and role. In French, the term ‘case manager’ does not automatically refer to someone who accompanies. Frequently, old people did not recognize their case manager when the investigator designated her by that title, but were able to identify her by her surname or even her first name. Others, for whom the name did not ring a bell, asked the investigator if he was talking about “that young”, “nice” or “small” woman, among the different people intervening at their homes. Thus, the old people showed evidence of a real personification of the case manager representing them as an individual and not a role. For some of them, the case manager’s role was not clear but some admitted: “I don’t really know, but she often comes to see me, asks questions, and we discuss.”

Some old people called the case manager a “social worker” and her role was assimilated with that function. This assimilation is explained by the three-dimensional view of those intervening in their homes: medical personnel involved with physical or medical treatments, among which they easily identify the doctor, physio-therapist and visiting nurse sometimes confused with the nurse’s aide. There are also “housekeepers” who comprise a group including caregivers and all the other professionals involved in meals and housekeeping. Finally, the “social workers” are all the people intervening in their homes belonging to the first two categories and who “take care of all the paperwork”.

#### A friend allaying loneliness

The frequency of case managers’ visits meant that they progressively became well-known faces, people whose visits the old people generally appreciated, even though their role was not yet completely understood. A minimum description was of the case manager as someone who came often and took the time to discuss and ask questions. The vocabulary ascribed to her was suggestive of friendship. “She is very agreeable. She’s an adorable young woman, very kind. We met and I told her: “you have become a true friend.”

The haziness as to the case managers’ real functions, in addition to this professional’s polyvalence, led old people to represent them as the person there to help in a general manner. The old people did not refer to their professional technical competencies but rather saw them as a resource person, in the same way they would for a non-professional, dynamic-and-resourceful assistant. They stated that the case-manager’s role was to “help others”, “respond to all the questions” and to be “aware of everything”. Sometimes, the case manager was described as an individual at their service, who would take the time to reply to requests that would be refused by all other professionals. Basing their relationship with the case manager on an affective level, the old people hoped to influence some of the essential aspects of the aid provided or even to manipulate the case manager. Hence, some of them, especially those refusing assistance, sought to make the case manager an ally to justify their refusal to undergo medical examinations, others tried to use her as a spokeswoman for the decisions that they did not dare assume on their own. This was the case for the user who wanted her case manager to reprimand and dismiss her housekeepers. What is more, the old people also considered the case manager as a professional whose duty was to do all that needed to be done, and made incongruous demands, failing to distinguish between their real needs and their punctual wants. When the case manager refused to succumb to these requests, they reacted by casting doubt on her goodwill and questioning the trust accorded her. “I asked her to go with me by taxi but she said nothing and left; she didn’t want to.”

The fact that the case manager often visits the user’s home and/or calls him/her regularly for updates contributed to the user representing her as a member of the domestic sphere, a sort of friendly helper taking the place that could be accorded a neighbour or member of the family. Old people were rarely aware of the actions taken by case manager outside the home, such as coordinating help. For them, the case manager’s duty was to be at their side at home and to respond to their demands. Some had the similar expectations of a case manager as of a neighbor or member of the family. Therefore, they asked for a visit or took the opportunity of a visit already programmed to request that she call the doctor or accompany them to the bakery.

This perception explains why old people with informal caregivers highly involved in their accompaniment, and often exhausted, did not consider a case manager’s presence and actions necessary. An old person whose accompaniment was assured by guardians explained: “we get it done quite well together, we don’t need anyone else.”

## Discussion

Our results are congruent with previous qualitative research which aiming to explore the role of the case manager through the eyes of the case managers themselves [7, 24] or through those of the old people [24]. The case managers we interviewed insisted on the role of “solver”, “supporter”, “guide”, “guard”, “navigator” and the necessity to gain the trust and the confidence of the users in order to perform their tasks of evaluation and care planning.

The main disparities we note with the Sandberg et al. [24] study concerning the old people’s point of view could be explained by the differences in our sample. Sandberg and al. included “cognitively adequate” participants with a MMSE cut-off of 25 points. This explains why in our study the representation of the case manager as a “solution finder” or “a partner” is mainly the one of the informal caregivers instead of the one of the old people interviewed by Sandberg.

By interviewing all the old people who wanted to participate without excluding some because of possible cognitive impairments, we could go deeper in the analysis of the discourse of those Sandberg designed as those who “did not know what the CM could do”. Our results show that if the these people are not able to describe precisely what the case manager did for them, most consider the case manager as a helper, a confident and an isolation breaker.

In the framework of a qualitative inductive protocol, this result is crucial as it is necessary to consider what is important for old people by including everything they said about the actions or advice of the case manager. Frail old people are not focused on what the case manager could bring them but on her presence, her ability to listen. For them the case manager should be someone able to defend their voice. Even if these points are difficult to include in the evaluation of the effectiveness of the case management service they are surely related to the quality of the interpersonal continuity and the advocacy mission.

There is a need to conduct qualitative inductive research and to collect and analyze the representations of the case manager’s roles during the implementation phase because these representations have an influence on the implementation process. Moreover, the first representations of the case manager’s role by users have also to be regarded as starting points for the evaluation protocol which aims to measure the impacts of the case management service.

Many studies [7, 24, 25] insisted on trust and confidence presenting them as crucial elements of case management intervention to enhance interpersonal continuity [26] but the way how trust and confidence are obtained are rarely discussed. In agreement with previous studies [27, 28], our study reveals that the user’s representations on the role of the case manager as “friend” have been for a means of securing trust and confidence. Whereas many old people refused care and informal caregivers felt isolated, the case manager was considered as different sort of helper. The fact that the fieldwork was done during the implementation phase – step during which the visits of case manager were frequent- has undoubtedly reinforced this point. The fact that the old people considered the case manager as a kind of “assisting friendship” [29] who visits us was a means of facilitating the entry into the home of the old people and the first step of interpersonal continuity.

In the French context, it appears that the kind of vagueness related to the perceived role of case managers and the limits of their interventions do not hinder implementation as it was shown in other contexts [30]. We consider that the vagueness expressed by users related to the role of the case manager is not only due to misunderstanding but due to adaptability and flexibility inherent in the role of case manager confronted with complex situations. The fact that users perceived the case manager as friend more than professional permits to outweigh the reluctance and the refusal users usually expressed toward the other professionals.

These conclusions prove the usefulness of our qualitative inductive analysis of the effects of the implementation of the case management service in revealing factors that were not necessarily foreseen and could not be evaluated by an instrumental evaluation of care satisfaction or a randomized control trials that focus on the reduction of unplanned hospitalization, psychological well-being or unmet service need [31].

Our results show the representation of the case managers by the informal caregivers was not fixed and evolved during the implementation. Moreover, it appears that their satisfaction with the service is closely linked with their idea of what should be the role of the case manager. This potentially explains why some studies [32] showed a decrease in satisfaction with the service for informal caregivers. We can hypothesize that the satisfaction decreases when the disparity between the expectations as to the role of case manager and the actions carried out become too great.

Thus the usefulness of considering informal caregiver satisfaction via a longitudinal approach taking into account representations and expectations with regard to the role of the case manager.

### The limitations of the study

The main limits of this study are linked to the objectives of our protocol which was to explore the representations of old people, their informal caregivers and the case managers during the implementation phase. The CM program was new in France and the word “case manager” was not known by the people involved in the program.

Representations are not set; in this case they changed as the CM service evolved. A longitudinal study would be necessary to follow the evolution of the representations and to better understand their links with the work of coordination done by the case managers.

The other point is that many old people involved in case management had refused care. This specific profile of the main users of the service had probably influenced the attitude of the case manager toward them and toward their informal caregiver and as a consequence, their representation of her role.

## Conclusion

In French, the word case manager is not well known that is why people build their own representations of what could be its role. Moreover, the PRISMA-France project consisted in a sort of pilot study in which the aim of the research part was first to analyze how the different participants (case managers, old people, informal caregivers) understand and adopt the service.

Our results show that the representations of case manager’s role are mainly influence by the analysis of the situation made by the different people involved. Case managers of focused on what they consider as their job: finding solutions destined to preserve the autonomy of old people. Thus, becoming the confident of old people appears as a necessary step in order to represent them. Confronted with the situation, informal caregivers feel distraught and perceived the case manager as the one who could help and relieve them. Finally, if many of the old do not really understand the case manager’s role, they are able to say they appreciate her presents and attention. Even if it is difficult to include it in the evaluation of the effectiveness of the case management service this is necessary to take in into account in respect to the quality of the interpersonal continuity and the advocacy mission.

Analyzing the first representations of the case manager’s role during the implementation phase of the CM service is a necessary step before considering the effects of the services. If we want to understand the effect of the CM services, the implementation and the success of a case management model have to be evaluated regarding the previous healthcare context and the expectations of people concerned. The case manager’s strength is precisely his/her flexibility and versatility giving him/her the ability to answer the complex needs of the old people they accompany.

## Abbreviations

CM: Case management
CPP: Comité de protection des personnes
GP: General practitioner
PRISMA: Projet et Recherches sur l’Intégration des Services pour le Maintien de l’Autonomie
